# Supplementary material for: TNF antagonist sensitizes synovial fibroblasts to ferroptotic cell death in collagen-induced arthritis mouse models
Source: Nat Commun. 2022 Feb 3;13:676. doi: 10.1038/s41467-021-27948-4 (PMC8813949; doi:10.1038/s41467-021-27948-4)
Supplement: Supplementary file 1 — Supplementary Information [file 41467_2021_27948_MOESM1_ESM.pdf]

## Supplementary Information

TNF Antagonist Sensitizes Synovial Fibroblasts to Ferroptotic Cell Death in Collagen-induced Arthritis Mouse Models

First Authors: Jiao Wu, Zhuan Feng, Liang Chen, Yong Li
Corresponding Authors: Jiao Wu, Xuejun Jiang, Zhi-Nan Chen & Ping Zhu

Supplementary Figures 1-12
Supplementary Tables 1-7

## Supplementary Figure 1

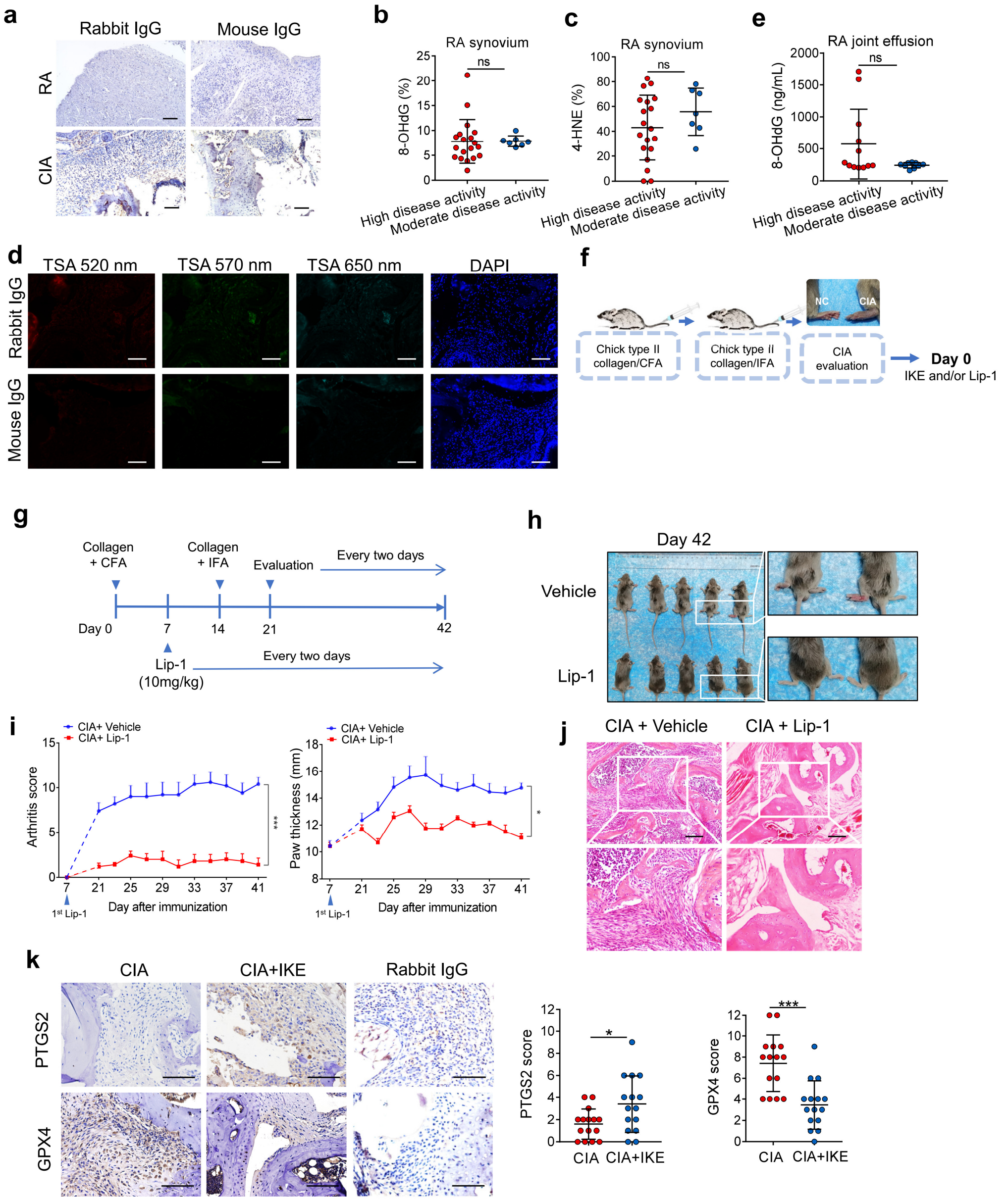


**Supplementary Fig. 1. IKE induces ferroptosis in fibroblast populations from the synovium of RA patients. a** Immunohistochemistry on formalin-fixed paraffin embedded human RA synovium or mouse CIA joints using 10 µg/mL of rabbit IgG1 isotype control or mouse IgG1 isotype control followed by an anti-mouse/rabbit avidin-biotin HRP detection system. Nuclei are counterstained with hematoxylin. Scale bars, 100 µm. The isotype control was repeated independently along with the staining of different molecules with similar results. **b** Quantitative comparison of 8-OHdG staining in the synovium of RA patients with different disease activities. High disease activity, n = 19 RA patients; moderate disease activity, n = 7 RA patients. ns, $P$ = 0.9846; two-tailed t test. **c** Quantitative comparison of 4-HNE staining in the synovium of RA patients with different disease activities. High disease activity, n = 19 RA patients; moderate disease activity, n = 7 RA patients. ns, $P$ = 0.2505; two-tailed t test. **d** Fluorescent multiplex IHC staining of mouse CIA joints using 10 µg/mL of rabbit IgG1 isotype control or mouse IgG1

isotype control followed by TSA detection kit. Scale bars, 200 μm. The isotype control was repeated independently along with the staining of different molecules with similar results. **e** 8-OHdG concentration in the joint fluid of RA patients with different disease activities. High disease activity, n = 12 RA patients; moderate disease activity, n = 8 RA patients. ns, *P* = 0.0853; two-tailed t test. **f** Establishment of a CIA mouse model and administration of IKE and/or Lip-1 treatment. **g** Establishment of a CIA mouse model and administration of Lip-1 treatment started before the shown up of significant phenotype of arthritis. **h** Representative images of the paws from mice with or without administration of Lip-1 42 days after immunization with type II collagen. **i** Joint inflammation measured by arthritis score (left) and paw thickness (right) in CIA model mice that were intraperitoneally injected with Lip-1 every two days for 5 weeks. ***$P$ = 0.0008, *$P$ = 0.0111; two-way repeated measure ANOVA. n = 5 mice for each group. **j** H&E staining images of representative joints in CIA model mice with or without administration of Lip-1. Scale bars, 100 μm. Similar results were observed from 15 joints tested for each group. **k** Immunohistochemical staining and scoring of PTGS2 and GPX4 in the joints of CIA model mice. n = 15 joints for each group. *$P$ = 0.0228, ***$P$ = 0.0002; two-tailed t test. Scale bars, 100 μm. Data in **i** are presented as mean ± SEM. Other data are presented as mean ± SD. Source data are provided as a Source Data file.

Supplementary Figure 2

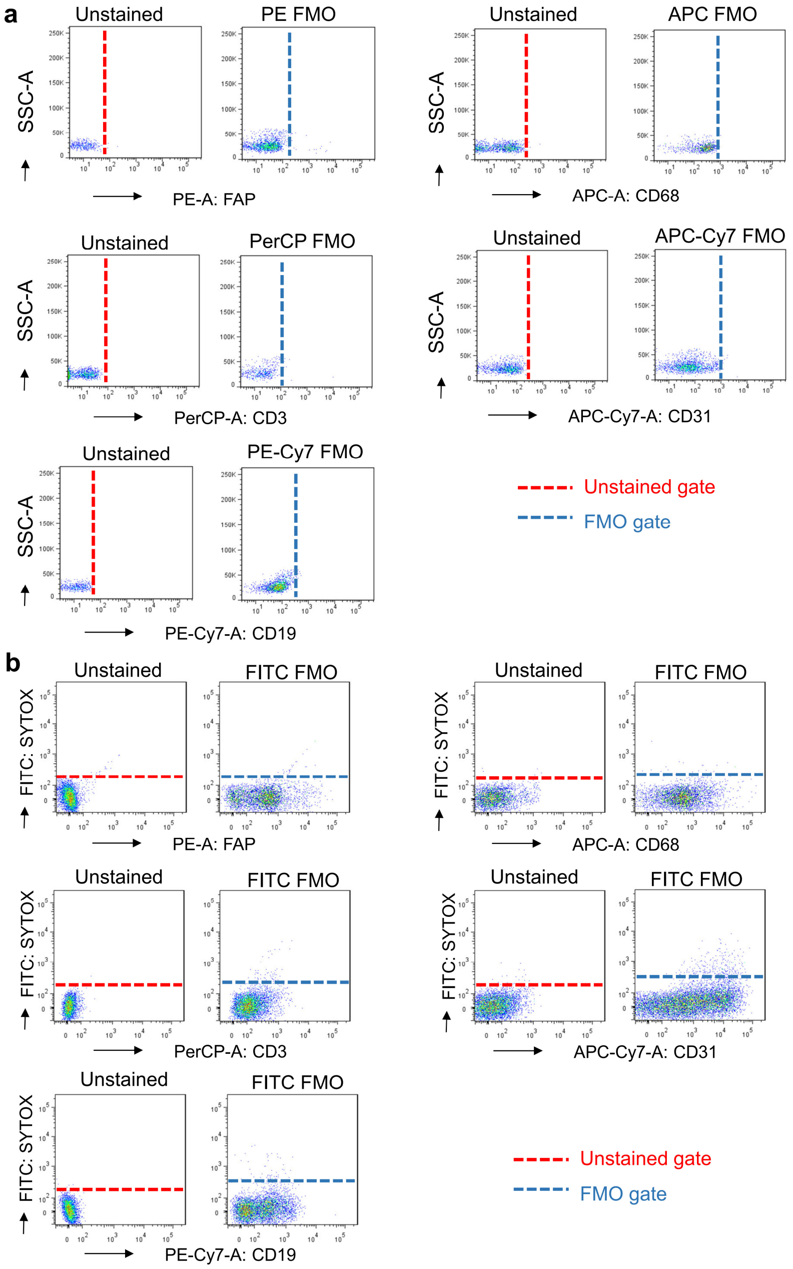


**Supplementary Fig. 2. FMO controls used in Fig. 2c. a** FMO for PE-FAPα, APC-CD68, PerCP-CD3, APC-Cy7-CD31 and PE-Cy7-CD19. **b** FMO for SYTOX Green.

## Supplementary Figure 3

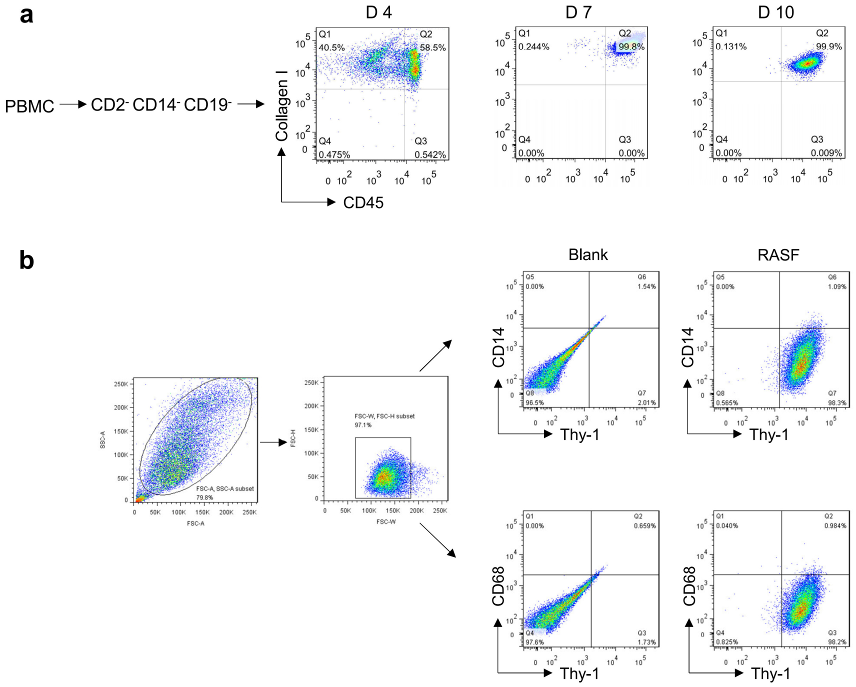


**Supplementary Fig. 3. Isolation and identification of circulating fibroblasts and synovial fibroblasts. a** CD2-CD14-CD19- cells from PBMCs of RA patients were cultured for the indicated times, and the percentages of CD45+ Collagen 1+ cells were analyzed by flow cytometry. **b** Flow cytometric analysis of digested synovial cells cultured from RA patients, and fibroblasts were identified as the CD14- CD68- Thy1+ population by flow cytometry.

Supplementary Figure 4

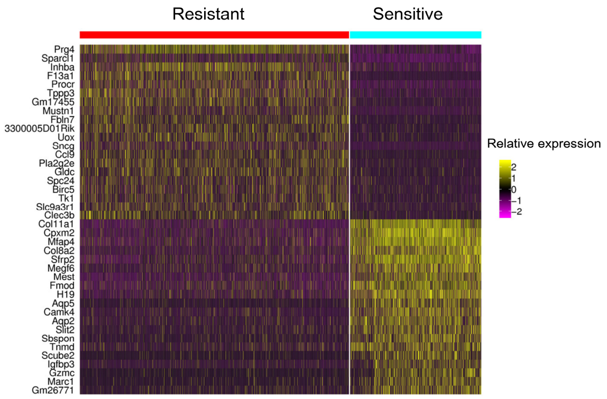


**Supplementary Fig. 4. Differential gene expression of ferroptosis-sensitive and ferroptosis-resistant fibroblast subsets.** The heat map shows the expression of the top 20 genes (by *P* value) discovered that were considered significant and conserved marker genes for each subset.

## Supplementary Figure 5

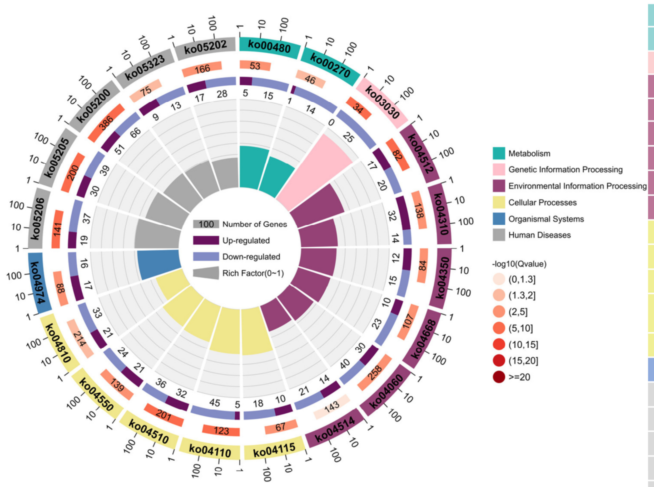


| ID | Description |
|---|---|
| ko00480 | Glutathione metabolism |
| ko00270 | Cysteine and methionine metabolism |
| ko03030 | DNA replication |
| ko04668 | TNF signaling pathway |
| ko04512 | ECM-receptor interaction |
| ko04310 | Wnt signaling pathway |
| ko04350 | TGF-beta signaling pathway |
| ko04060 | Cytokine-cytokine receptor interaction |
| ko04060 | Cell adhesion molecules (CAMs) |
| ko04115 | p53 signaling pathway |
| ko04110 | Cell cycle |
| ko04510 | Focal adhesion |
| ko04550 | Signaling pathways regulating pluripotency of stem cells |
| ko04810 | Regulation of actin cytoskeleton |
| ko04974 | Protein digestion and absorption |
| ko05206 | MicroRNAs in cancer |
| ko05205 | Proteoglycans in cancer |
| ko05200 | Pathways in cancer |
| ko05323 | Rheumatoid arthritis |
| ko05202 | Transcriptional misregulation in cancers |

**Supplementary Fig. 5. KEGG (Kyoto Encyclopedia of Genes and Genomes) enrichment analysis of differentially expressed genes in ferroptosis-sensitive and ferroptosis-resistant fibroblast subsets.** The first lap indicates the KEGG pathway; the second lap indicates the number of genes in the genome background and -log10 Q values for enrichment of the differentially expressed genes for the KEGG pathway; the third lap indicates the ratio of the upregulated genes (purple) and downregulated genes (blue); and the fourth lap indicates the rich factor of each pathway.

Supplementary Figure 6

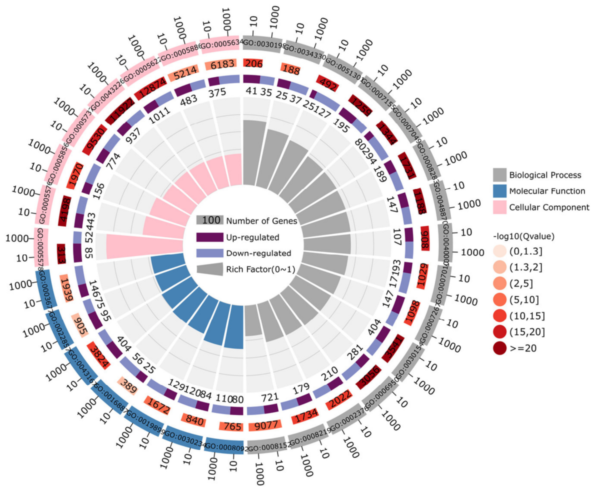


| ID | Description |
|---|---|
| GO:0008219 | cell death |
| GO:0006950 | response to stress |
| GO:0008283 | cell proliferation |
| GO:0051301 | cell division |
| GO:0040007 | growth |
| GO:0007049 | cell cycle |
| GO:0007155 | cell adhesion |
| GO:0048870 | cell motility |
| GO:0030198 | extracellular matrix organization |
| GO:0034330 | cell junction organization |
| GO:0007010 | cytoskeleton organization |
| GO:0007267 | cell-cell signaling |
| GO:0008152 | metabolic process |
| GO:0002376 | immune system process |
| GO:0030154 | cell differentiation |
| GO:0043167 | ion binding |
| GO:0008092 | cytoskeletal protein binding |
| GO:0030234 | enzyme regulator activity |
| GO:0019899 | enzyme binding |
| GO:0003677 | DNA binding |
| GO:0016887 | ATPase activity |
| GO:0022857 | transmembrane transporter activity |
| GO:0005576 | extracellular region |
| GO:0005578 | proteinaceous extracellular matrix |
| GO:0043226 | organelle |
| GO:0005622 | intracellular |
| GO:0005737 | cytoplasm |
| GO:0005856 | cytoskeleton |
| GO:0005634 | nucleus |
| GO:0005886 | plasma membrane |

**Supplementary Fig. 6. GO (Gene Ontology) enrichment analysis of differentially expressed genes in ferroptosis-sensitive and ferroptosis-resistant fibroblast subsets.**

## Supplementary Figure 7

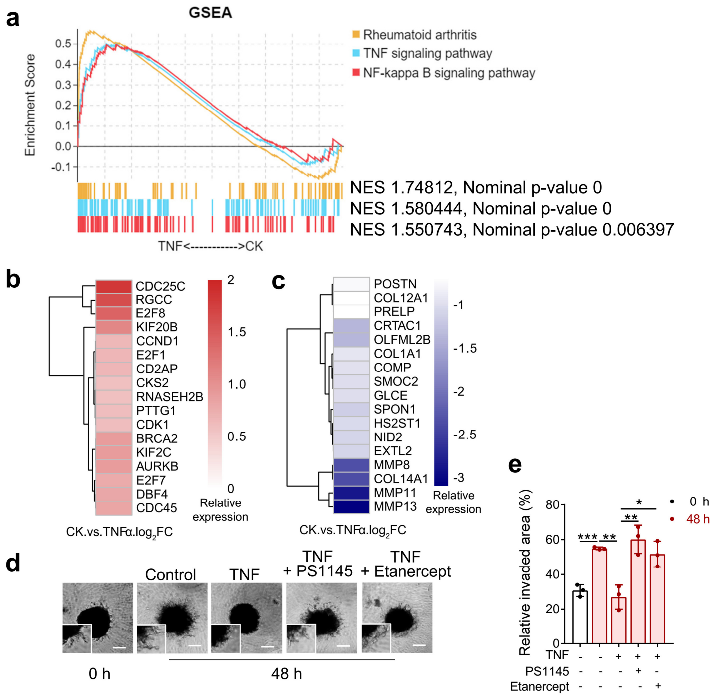


**Supplementary Fig. 7. Long-term exposure of fibroblasts to TNF increases the proliferation potential and decreases the genes related to extracellular matrix in fibroblasts. a-c** Fibroblasts were stimulated with TNF (20 ng/ml) for 96 h, and RNA sequencing was performed. **a** Enrichment plots of GO_Rheumatoid, TNF signaling pathway and NF-κB signaling pathway gene sets among TNF-stimulated fibroblasts compared to control fibroblasts as identified by GSEA. The p-value is calculated through permutation tests. **b** Genes enriched in the GO_Cell division gene set (left) and KEGG_DNA replication gene set among the ferroptosis-resistant subset were cross-referenced against genes that showed increased expression after TNF treatment of fibroblasts from individuals with RA, and the expression of selected mRNA transcripts is shown. **c** Genes enriched in the GO_Proteinaceous extracellular matrix gene set (left) and KEGG_Glycosaminoglycan biosynthesis gene set among the ferroptosis-sensitive subset were cross-referenced against genes that were decreased after treatment of RA fibroblasts with TNF, and the expression of selected mRNA transcripts is shown. **d** Representative images of fibroblast spheroids grown in Matrigel and treated with TNF, PS1145 and etanercept for the indicated times. Scale bars, 100 μm. **e** The protrusion area extruded from the main body of the spheroid was monitored. n = 3 biologically independent samples per condition. ****P* = 0.0003; ***P* = 0.0023, 0.0058 (left to right); **P* = 0.0135; two-tailed t-test. All data are presented as mean ± SD. Source data are provided as a Source Data file.

## Supplementary Figure 8

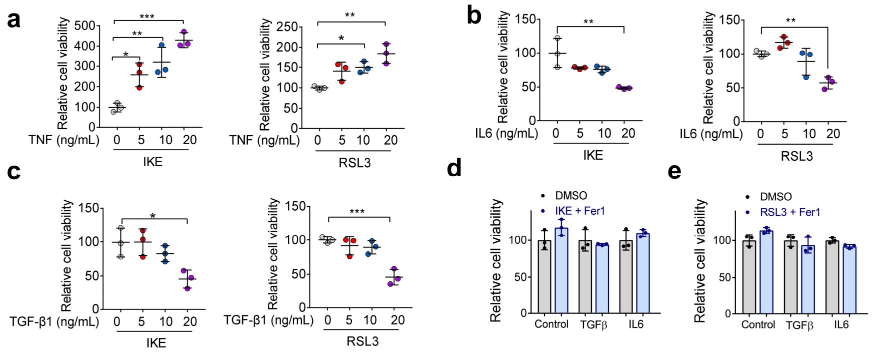


**Supplementary Fig. 8. TNF protects fibroblasts against ferroptosis, while IL-6 and TGF-β sensitize fibroblasts to ferroptosis. a-c** Relative viability of fibroblasts from individuals with RA that are primed with different concentrations of TNF (**a**), IL-6 (**b**) and TGF-β (**c**), followed by treatment with IKE (1 μM, 28 h) or RSL3 (0.125 μM, 14 h). n = 3 biologically independent samples per condition. **a** Left, * $P$ = 0.0231, **$P$ = 0.0035, ***$P$ = 0.0002. Right, * $P$ = 0.0384, **$P$ = 0.0020; one-way ANOVA followed by multiple comparisons. **b** Left, **$P$ = 0.0022. Right, **$P$ = 0.0097; one-way ANOVA followed by multiple comparisons. **c** Left, *$P$ = 0.0205. Right, ***$P$ = 0.0009; one-way ANOVA followed by multiple comparisons. **d-e** Relative viability of fibroblasts primed with TGF-β or IL-6, followed by treatment with IKE (**d**) or RSL3 (**e**) in the presence of Fer1. n = 3 biologically independent samples per condition. All data are presented as mean ± SD. Source data are provided as a Source Data file.

Supplementary Figure 9

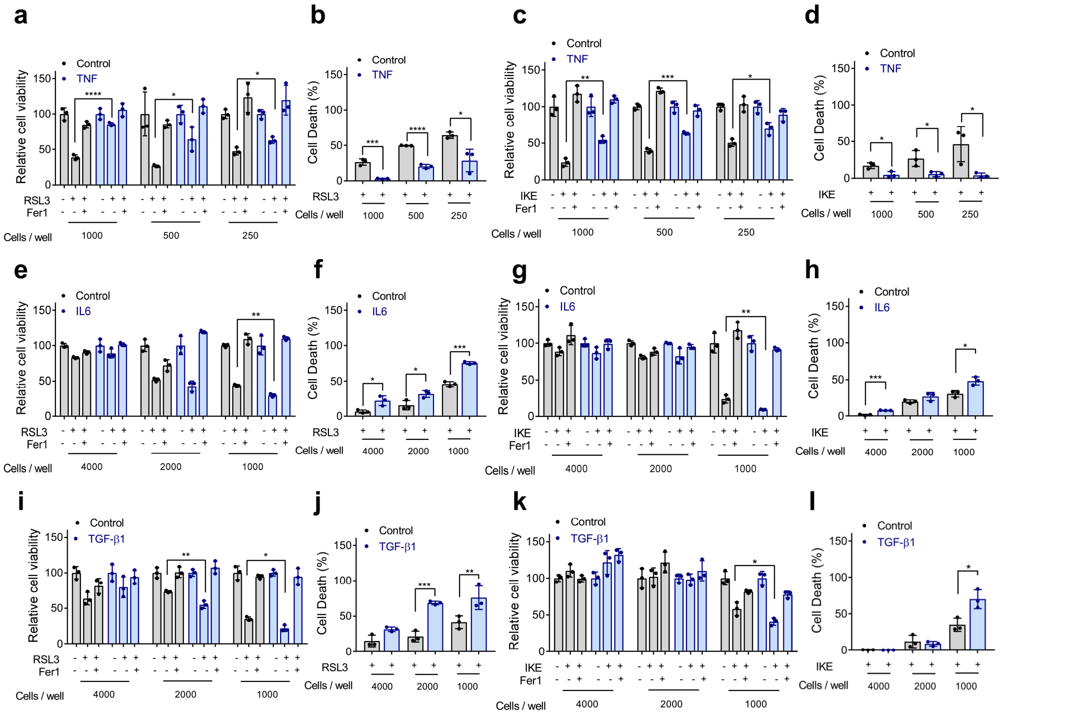


**Supplementary Fig. 9. TNF reduces ferroptosis susceptibility in a cell density-independent manner. a-d** Fibroblasts from individuals with RA were seeded at the indicated density in 96-well plates and primed with TNF. Relative viability was measured after RSL3 treatment for 12 h (**a**) or IKE treatment for 26 h (**c**), and cell death was measured after RSL3 treatment for 10 h (**b**) or IKE treatment for 24 h (**d**). Cells were stained with hoechst 33342 (0.1 μg/mL) to monitor total cell number, and with SYTOX green (5 nM) to monitor cell death. **a** ****$P < 0.0001$, *$P = 0.0194$, 0.0171 (left to right); two-tailed t-test. **b** *** $P = 0.0009$, ****$P < 0.0001$, *$P = 0.0198$; two-tailed t-test. **c** ** $P = 0.0018$, ***$P = 0.0002$, *$P = 0.0280$; two-tailed t-test. **d** *$P = 0.0215$, 0.0325, 0.0382 (left to right); two-tailed t-test. **e-h** Fibroblasts from individuals with RA and set at the indicated density were primed with IL-6 and then treated with RSL3 or IKE, and relative cell viability and cell death were measured as described above. **e** **$P = 0.0010$; two-tailed t-test. **f** *$P = 0.0217$, 0.0277 (left to right), *** $P = 0.0002$; two-tailed t-test. **g** ** $P = 0.0012$; two-tailed t-test. **h** ***$P = 0.0003$, *$P = 0.0115$; two-tailed t-test. **i-l** Fibroblasts from individuals with RA and set at the indicated density were primed with TGF-β and then treated with RSL3 or IKE, and relative cell viability and cell death were measured as described above. **i** **$P = 0.0045$, *$P = 0.0107$; two-tailed t-test. **j** ***$P = 0.0005$, ** $P = 0.0021$; two-tailed t-test. **k** * $P = 0.0465$; two-tailed t-test. **l** *$P = 0.0182$; two-tailed t-test. n = 3 biologically independent samples per condition. All data are presented as mean ± SD. Source data are provided as a Source Data file.

## Supplementary Figure 10

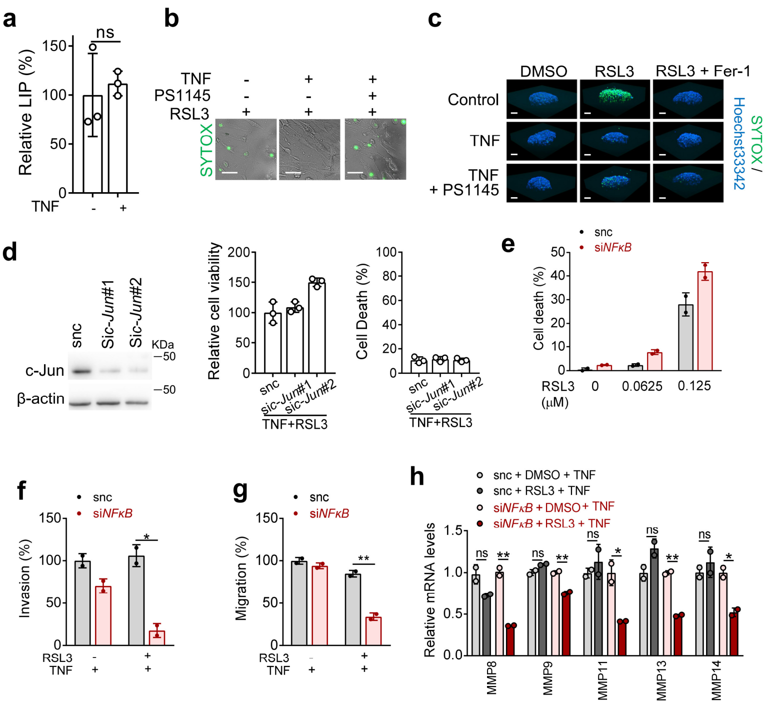

**Supplementary Fig. 10. Sublethal doses of ferroptosis-inducers modulates the aggressive behaviors of RA fibroblasts. a** Cellular labile iron pool (LIP) level in fibroblasts treated with TNF. n = 3 biologically independent samples per condition. ns, $P = 0.6721$; two-tailed t test. **b** Microscopy showing cell death. Scale bars, 20 µm. Similar results were observed from 3 times independent experiments. **c** Three-dimensional spheroids formed by fibroblasts were treated as indicated. Dead cells were stained with SYTOX green, while all nuclei were stained with Hoechst 33342 (blue). Scale bars, 100 µm. **d** Left, western blotting analysis of c-Jun expression in fibroblasts transfected with scramble siRNA or either of two independent siRNAs targeting c-Jun, which is a component of AP-1 (sic-*Jun*#1, sic-*Jun*#2). Right, relative viability and cell death of fibroblasts transfected with scramble siRNA or siRNA targeting c-Jun and treated with TNF followed by RSL3. n = 3 biologically independent samples per condition. **e** Cell death of fibroblasts transfected with scramble siRNA or siRNA targeting NF-κB p65, and then treated with indicated concentrations of RSL3. n = 2 biologically independent samples per condition. **f-g** Invasive (**f**) and migrative (**g**) potentials of RA fibroblasts transfected with scramble siRNA or siRNA targeting NF-κB p65, and then treated with TNF followed by RSL3 (0.0625 µM), measured by the transwell assay. n = 2 biologically independent samples per condition. $^{*}P$ = 0.0147, $^{**}P$ = 0.0065; two-tailed t test. **h** The mRNA levels of MMP8, -9, -11, -13 and -14 in NF-κB p65 knockdown fibroblasts with TNF and RSL3 treatment. n = 2 biologically independent samples per condition. ns, $P$ = 0.0911, 0.0644, 0.4768, 0.1108, 0.4827 (left to right); $^{*}P$ = 0.0324, 0.0195 (left to right); $^{**}P$ = 0.0049, 0.0072, 0.0015 (left to right); two-tailed t test. All data are presented as mean ± SD. Source data are provided as a Source Data file.

Supplementary Figure 11

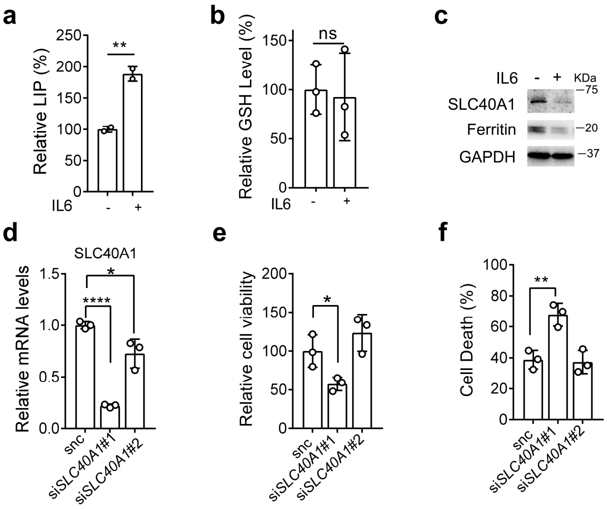


**Supplementary Fig. 11. IL-6 sensitized fibroblasts to ferroptosis. a** The LIP level in fibroblasts treated with IL-6. n = 2 biologically independent samples per condition. ***P* = 0.0098; two-tailed t test. **b** Intracellular GSH in fibroblasts treated with IL-6 for 72 h. n = 3 biologically independent samples per condition. ns, *P* = 0.8078; two-tailed t test. **c** Western blotting analysis of SLC40A1 and Ferritin expression in fibroblasts stimulated with IL-6 for 72 h. **d** Relative mRNA levels of SLC40A1 in fibroblasts transfected with scramble siRNA or either of two independent siRNAs targeting SLC40A1 (siSLC40A1#1, siSLC40A1#2). n = 3 biologically independent samples per condition. **P* = 0.0162, *****P* < 0.0001; one-way ANOVA followed by multiple comparisons. **e-f** Relative viability (**e**) and cell death (**f**) of fibroblasts transfected with scramble siRNA or siRNA targeting SLC40A1and treated with IL-6 followed by RSL3. n = 3 biologically independent samples per condition. **P* = 0.0312, ***P* = 0.0063; two-tailed t test. All data are presented as mean ± SD. Source data are provided as a Source Data file.

## Supplementary Figure 12

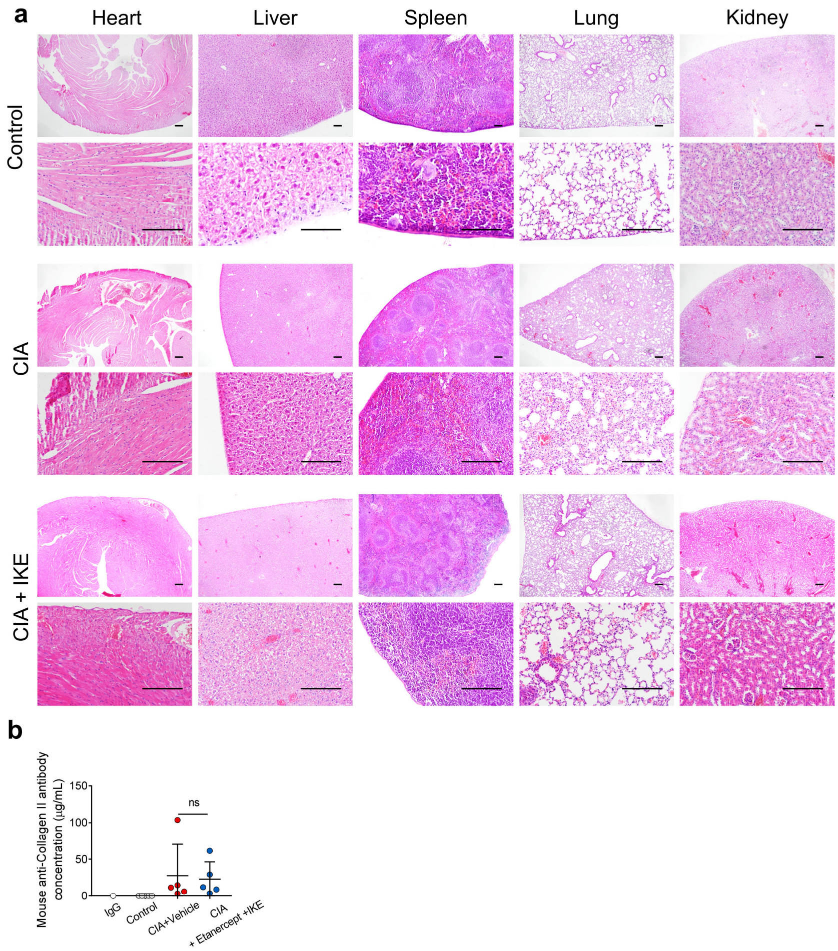


**Supplementary Fig. 12. Pathological changes in organs of CIA mice treated with IKE. a** H&E staining images of representative hearts, livers, spleens, lungs and kidneys in control and CIA mice treated with or without IKE (20 mg/kg, twice a week) for 36 days. Scale bars, 200 μm. Similar results were observed from all 5 mice tested for each group. **b** Detection of anti-type II collagen antibody in serum of CIA mice treated with etanercept in combination with IKE, performed with ELISA. n = 5 biologically independent animals per group. ns, $P = 0.9612$; two-tailed t test. Data are presented as mean ± SD. Source data are provided as a Source Data file.

Supplementary Table 1. Characteristics of rheumatoid arthritis (RA) individuals and osteoarthritis (OA) individuals for immunohistochemistry studies.

| Characteristics | RA | OA |
|---|---|---|
| Number of patients | 26 | 21 |
| Age in years, median (IQR) | 48.0 (39.5-54.3) | 55.0 (49.5-57.0) |
| Female sex, (%) | 14 (53.8) | 12 (57.1) |
| Disease duration, year, median (IQR) | 11 (6.8-15.0) | 12 (7.0-15.0) |
| ESR, mm/hour, median (IQR) | 52.5 (21.50-71.25) | 19.0 (8.50-33.50) |
| DAS28, median (IQR) | 5.76 (4.25-6.04) | na |

Values are presented as median (interquartile range) or number (percentage). IQR, interquartile range, ESR, erythrocyte sedimentation rate; DAS28, 28-joint disease activity score; na, not applicable.

Supplementary Table 2. Characteristics of RA individuals for detection of MDA, 8-OHdG and iron levels in joint fluid.

| Characteristics | RA |
|---|---|
| Number of patients | 20 |
| Age in years, median (IQR) | 53.0 (50.0-61.3) |
| Female sex, (%) | 16 (80.0) |
| Disease duration, year, median (IQR) | 4.5 (1.1-9.8) |
| High diseases activity, (%) | 12 (60) |
| Moderate diseases activity, (%) | 8 (40) |
| ESR, mm/hour, median (IQR) | 43.5 (31.25-56.25) |
| DAS28, median (IQR) | 6.22 (4.85-6.43) |

Values are presented as median (interquartile range) or number (percentage). IQR, interquartile range; ESR, erythrocyte sedimentation rate; DAS28, 28-joint disease activity score.

Supplementary Table 3. Characteristics of RA individuals for single-cell RNA sequencing.

| Characteristics | RA |
|---|---|
| Number of patients | 5 |
| Age in years, average | 49.8 |
| Female sex, (%) | 5 (100) |
| Disease duration, year, average | 7.8 |
| ESR, mm/hour, average | 38.2 |
| DAS28, average | 6.61 |

Values are presented as average or number (percentage). ESR, erythrocyte sedimentation rate; DAS28, 28-joint disease activity score.

Supplementary Table 4. Characteristics of RA individuals for isolation of synovial fibroblasts from joint fluid and circulating fibrocytes from PBMC.

| Characteristics | RA |
|---|---|
| Number of patients | 6 |
| Age in years, average | 48.3 |
| Female sex, (%) | 4 (66.7) |
| Disease duration, year, average | 0.7 |
| ESR, mm/hour, average | 47.17 |
| DAS28, average | 5.54 |

Values are presented as average or number (percentage). ESR, erythrocyte sedimentation rate; DAS28, 28-joint disease activity score.

Supplementary Table 5. The sequences of siRNA.

| Names of siRNA primers | Sequences of primers |
|---|---|
| SLC40A1#1-sense | GGUGAGUGUUGUUAUAUAAUU |
| SLC40A1#1-antisense | UUAUAUAACAACA-CUCACCUU |
| SLC40A1#2-sense | GAGAGACUCYAGAAGUUAAUU |
| SLC40A1#2-antisense | UUAACUUCUAGAGUCUCUCCU |
| GCLC#1-sense | GCUAAUGAGUCUGACCAUUdTdT |
| GCLC#1-antisense | AAUGGUCAGACUCAUUAGC |
| GCLC#2-sense | GUAGUAUUCUGAACUACCUdTdT |
| GCLC#2-antisense | AGGUAGUUCAGAAUAUAUCdTdT |
| p65 #1-sense | GGACAUAUGAGACCUUCAAdTdT |
| p65 #1-antisense | UUGAAGGUCUCAUAUGUCCdTdT |
| p65 #2- sense | GAUGAGAUCUUCCUACUGUdTdT |
| p65 #2- antisense | ACAGUAGGAAGAUCUCAUdTdT |
| c-Jun #1- sense | CAGUGUUGUUUGGUAAAUAAGA |
| c-Jun #1- antisense | UUAUUUACAAACAACACUGGG |
| c-Jun #2- sense | GCUGAUUACUGUCAAUAAACA |
| c-Jun #2- antisense | UUUAUUGACAGUAAUCAGCUU |
| Negative Control sense | UUC UCC GAA CGU GUC ACG UTT |
| Negative Control antisense | ACG UGA CAC GUU CGG AGA ATT |

Supplementary Table 6. The sequences of PCR primers.

| **Names of PCR primers** | **Sequences of PCR primers** |
|---|---|
| huSLC40A1-Forward Primer | TGGATGGGTTCTCACTTCCTG |
| huSLC40A1-Reverse Primer | GTCAATCCTTCGTATTGTGGCAT |
| huMMP8 -Forward Primer | AAGTGGGAACGCACTAACTTG |
| huMMP8-Reverse Primer | GGATTCCATTGGGTCCATCAAAT |
| huMMP9-Forward Primer | TTCCCCTTCACTTTCCTGGGTA |
| huMMP9-Reverse Primer | CGCCACGAGGAACAAACTGTAT |
| huMMP11-Forward Primer | CCGCAACCGACAGAAGAGG |
| huMMP11-Reverse Primer | ATCGCTCCATACCTTTAGGGC |
| huMMP13-Forward Primer | TCTTCGGCTTAGAGGTGACTG |
| huMMP13-Reverse Primer | CAGAGGAGTTACATCGGACCA |
| huMMP14-Forward Primer | CATCTGTGACGGGAACTTTGA |
| huMMP14-Reverse Primer | GGCAGTGTTGATGGACGCA |
| GAPDH-Forward Primer | GCACCGTCAAGGCTGAGAAC |
| GAPDH-Reverse Primer | TGGTGAAGACGCCAGTGGA |

Supplementary Table 7. Resource information for antibodies.

| Antibody | Company | Cat# | Application | Dilution |
|---|---|---|---|---|
| FAP | Invitrogen | BMS168 | TSA-based immunofluorescent multiplex | 1:100 |
| FAPα | Abcam | ab218164 | TSA-based immunofluorescent multiplex | 1:100 |
| Mfap4 | Thermo | PA5-24865 | TSA-based immunofluorescent multiplex | 1:100 |
| Sparcl1 | Santa Cruz | sc-514275 | TSA-based immunofluorescent multiplex | 1:100 |
| F4/80 | Cell Signaling Technology | #70076 | TSA-based immunofluorescent multiplex | 1:500 |
| PerCP anti-CD3 | Biolegend | 317338 | Flow cytometric analysis | 5μl/test |
| PE anti-CD45 | Biolegend | 304008 | Flow cytometric analysis | 5μl/test |
| APC/Cyanine7 CD31 | Biolegend | 303120 | Flow cytometric analysis | 5μl/test |
| APC anti-CD68 | Biolegend | 333810 | Flow cytometric analysis | 5μl/test |
| PE/Cyanine7 anti-CD19 | Biolegend | 302216 | Flow cytometric analysis | 5μl/test |
| PE Goat anti-mouse IgG | Biolegend | 405307 | Flow cytometric analysis | 5μl/test |
| PE anti-human CD14 | Biolegend | 367104 | Flow cytometric analysis | 5μl/test |
| FAP | Invitrogen | BMS168 | Flow cytometric analysis | 1:100 |
| FITC anti-human CD90 (Thy1) | Biolegend | 328107 | Flow cytometric analysis | 5μl/test |
| FITC anti-Collagen I | Sigma-Aldrich | FCMAB412F | Flow cytometric analysis | 5μl/test |
| Dynabeads anti-CD19 | Invitrogen | 11143D | Isolation of circulating fibrocytes | Depletion: 50μl beads/2.5 x $10^7$ cells/ml |
| Dynabeads anti-CD2 | Invitrogen | 11159D | Isolation of circulating fibrocytes | Depletion: 50μl beads/2.5 x $10^7$ cells/ml |
| Dynabeads anti-CD14 | Invitrogen | 11149D | Isolation of circulating fibrocytes | Depletion: 50μl beads/2.5 x $10^7$ cells/ml |
| SLC40A1 | Abcam | ab78066 | Western blotting | 1:200 |
| GCLM | HuaBio | ET1705-87 | Western blotting | 1:500 |

| | | | | |
|---|---|---|---|---|
| GCLC | HuaBio | ET1704-38 | Western blotting | 1:500 |
| IκBα | HuaBio | ET1603-6 | Western blotting | 1:500 |
| phospho-IκBα | HuaBio | ET1609-78 | Western blotting | 1:500 |
| NF-kB p65 | HuaBio | ET1603-12 | Western blotting | 1:500 |
| SLC7A11 | Abcam | ab175186 | Western blotting | 1:1000 |
| c-Jun | Proteintech | 24909-1-AP | Western blotting | 1:1000 |
| ferritin | Abcam | ab75973 | Western blotting | 1:1000 |
| GAPDH | HuaBio | R1210-1 | Western blotting | 1:2000 |
| β-actin | HuaBio | R1102-1 | Western blotting | 1:2000 |
| 4-HNE | Abcam | ab48506 | Immunohistochemistry assay | 1:25 |
| 4-HNE | Abcam | ab46545 | Immunohistochemistry assay | 1:50 |
| PTGS2 | Abcam | ab179800 | Immunohistochemistry assay | 1:500 |
| GPX4 | Abcam | ab125066 | Immunohistochemistry assay | 1:50 |
| FAPα | Abcam | ab53066 | Immunohistochemistry assay | 1:50 |
| GCLC | Abcam | ab53179 | Immunohistochemistry assay | 1:100 |
| GCLM | Abcam | ab126704 | Immunohistochemistry assay | 1:50 |
| p-NF-κB p65 | Thermo Fisher | 44-711G | Immunohistochemistry assay | 1:50 |
| 8-OHdG | Abcam | ab48508 | Immunohistochemistry assay | 1:100 |
| VCAM-1 | Abcam | ab134047 | TSA-based immunofluorescent multiplex | 1:500 |
| 4-HNE | Abcam | ab46545 | TSA-based immunofluorescent multiplex | 1:50 |
| CD248 | Abcam | ab217535 | TSA-based immunofluorescent multiplex | 1:200 |
| TNFR1 | Sino Biological | 10872-R111 | blocking | 1:400 |
| TNFR2 | Sino Biological | 10417-R00N6 | blocking | 1:200 |
| rabbit IgG Isotype Control | Invitrogen | 31235 | IgG Isotype Control | 1:100 |
| mouse IgG Isotype Control | Invitrogen | 14-4714-82 | IgG Isotype Control | 1:100 |
