# Supplementary material for: TNF antagonist sensitizes synovial fibroblasts to ferroptotic cell death in collagen-induced arthritis mouse models
Source: Nat Commun. 2022 Feb 3;13:676. doi: 10.1038/s41467-021-27948-4 (PMC8813949; doi:10.1038/s41467-021-27948-4)
Supplement: Supplementary file 3 — Reporting Summary [file 41467_2021_27948_MOESM3_ESM.pdf]

nature research

Corresponding author(s): Ping Zhu

Last updated by author(s): Dec 6, 2021

Reporting Summary

Nature Research wishes to improve the reproducibility of the work that we publish. This form provides structure for consistency and transparency in reporting. For further information on Nature Research policies, see our Editorial Policies and the Editorial Policy Checklist.

Statistics

For all statistical analyses, confirm that the following items are present in the figure legend, table legend, main text, or Methods section.

n/a	Confirmed	
☐	☒	The exact sample size (*n*) for each experimental group/condition, given as a discrete number and unit of measurement
☐	☒	A statement on whether measurements were taken from distinct samples or whether the same sample was measured repeatedly
☐	☒	The statistical test(s) used AND whether they are one- or two-sided<br>*Only common tests should be described solely by name; describe more complex techniques in the Methods section.*
☐	☒	A description of all covariates tested
☐	☒	A description of any assumptions or corrections, such as tests of normality and adjustment for multiple comparisons
☐	☒	A full description of the statistical parameters including central tendency (e.g. means) or other basic estimates (e.g. regression coefficient) AND variation (e.g. standard deviation) or associated estimates of uncertainty (e.g. confidence intervals)
☐	☒	For null hypothesis testing, the test statistic (e.g. *F*, *t*, *r*) with confidence intervals, effect sizes, degrees of freedom and *P* value noted<br>*Give P values as exact values whenever suitable.*
☒	☐	For Bayesian analysis, information on the choice of priors and Markov chain Monte Carlo settings
☒	☐	For hierarchical and complex designs, identification of the appropriate level for tests and full reporting of outcomes
☐	☒	Estimates of effect sizes (e.g. Cohen's *d*, Pearson's *r*), indicating how they were calculated

Our web collection on statistics for biologists contains articles on many of the points above.

Software and code

Policy information about availability of computer code

Data collection	BD FACSDiVa™ software was used to collect data from flow cytometry. Inveon Acquisition Workplace from SIEMENS was used to collect micro-CT images. CellRanger(version 3.1.0) and NoveSeq 6000 (Illumina) were used to collect raw human single-cell RNA sequencing data.
Data analysis	Statistical analyses were performed with the GraphPad Prism 7.0 software (GraphPad). BD FACSDiva Software and Flowjo Software were used to analyze the data from flow cytometry. Raw gene expression matrices generated per sample in scRNA seq using CellRanger (version 3.1.0) were combined in R (version 3.5.3) mapping to GRCh38, converted to a Seurat object using the Seurat R package (version 3.0.1). Further data analysis was performed using Seurat R package, Gene Set Enrichment Analysis (GSEA) software, Gene Set Variation Analysis (GSVA) software, and CellPhoneDB. These software and code for scRNA seq are all open source, and the links have been provided in the nr-software-policy file. Quantitative analysis for immunohistochemistry assay and immunofluorescent multiplex was performed with Image-Pro Plus (Version 6.0) softwareSoftware or HALO™ Image Analysis Software.

For manuscripts utilizing custom algorithms or software that are central to the research but not yet described in published literature, software must be made available to editors and reviewers. We strongly encourage code deposition in a community repository (e.g. GitHub). See the Nature Research guidelines for submitting code & software for further information.

Data

Policy information about availability of data

All manuscripts must include a data availability statement. This statement should provide the following information, where applicable:

- Accession codes, unique identifiers, or web links for publicly available datasets
- A list of figures that have associated raw data
- A description of any restrictions on data availability

The scRNA-seq and RNA-seq data generated in this study have been deposited in the NCBI Sequence Read Archive (SRA) database under the accession code

PRJNA725073 [https://www.ncbi.nlm.nih.gov/bioproject/PRJNA725073] and PRJNA769634 [https://www.ncbi.nlm.nih.gov/sra/PRJNA769634]. The Croft et al. data used in this study are available in the GEO database under accession code GSE129087. All the source data and uncropped scans of blots supporting the findings of this study are provided with this paper as Source Data file. All codes have been deposited on Github (https://github.com/biolchen/TNF-and-Ferroptotic-Cell-Death).

Field-specific reporting

Please select the one below that is the best fit for your research. If you are not sure, read the appropriate sections before making your selection.

☒ Life sciences ☐ Behavioural & social sciences ☐ Ecological, evolutionary & environmental sciences

For a reference copy of the document with all sections, see nature.com/documents/nr-reporting-summary-flat.pdf

Life sciences study design

All studies must disclose on these points even when the disclosure is negative.

Sample size	Sample size is indicated in the figure legend for each experiments. For cell-based quantitative experiments, results of two or three independent biological replicates were used. In mouse experiments, we used mouse numbers that are small but sufficient for power calculation. The level of significance was set at 5%, and the power was set at 80%. The least practicable difference between groups was set at no less than 25%. Generally, the expected coefficient of variation was 15%. The group size is about 6 animals/group for a 4-group experiment. Based on our preliminary experiments, we analyze a sufficient number of animals per group (minimum 5 animals) to evaluate differences between different groups.
Data exclusions	No data exclusion
Replication	After conditions have been established, presented experiments were repeated for three times independently with reproducible results.
Randomization	For cell-based experiments, samples were randomly allocated to each group, and subsequently handled and processed identically. No potential covariates could be identified that required any additional controls. For animal experiments, animals were randomized for testing different conditions.
Blinding	The researcher performing the animal experiment was blind for the animal groups. The groups were code labeled other than labeled by treatments, which were reveled after results analysis. Another researcher was blinded when collecting measurements in the animal experiments. For in vitro cell culture studies blinding was not relevant to this study as all measures were quantifiable by standard cellular or biochemical assays.

Behavioural & social sciences study design

All studies must disclose on these points even when the disclosure is negative.

Study description	*Briefly describe the study type including whether data are quantitative, qualitative, or mixed-methods (e.g. qualitative cross-sectional, quantitative experimental, mixed-methods case study).*
Research sample	*State the research sample (e.g. Harvard university undergraduates, villagers in rural India) and provide relevant demographic information (e.g. age, sex) and indicate whether the sample is representative. Provide a rationale for the study sample chosen. For studies involving existing datasets, please describe the dataset and source.*
Sampling strategy	*Describe the sampling procedure (e.g. random, snowball, stratified, convenience). Describe the statistical methods that were used to predetermine sample size OR if no sample-size calculation was performed, describe how sample sizes were chosen and provide a rationale for why these sample sizes are sufficient. For qualitative data, please indicate whether data saturation was considered, and what criteria were used to decide that no further sampling was needed.*
Data collection	*Provide details about the data collection procedure, including the instruments or devices used to record the data (e.g. pen and paper, computer, eye tracker, video or audio equipment) whether anyone was present besides the participant(s) and the researcher, and whether the researcher was blind to experimental condition and/or the study hypothesis during data collection.*
Timing	*Indicate the start and stop dates of data collection. If there is a gap between collection periods, state the dates for each sample cohort.*
Data exclusions	*If no data were excluded from the analyses, state so OR if data were excluded, provide the exact number of exclusions and the rationale behind them, indicating whether exclusion criteria were pre-established.*
Non-participation	*State how many participants dropped out/declined participation and the reason(s) given OR provide response rate OR state that no participants dropped out/declined participation.*
Randomization	*If participants were not allocated into experimental groups, state so OR describe how participants were allocated to groups, and if allocation was not random, describe how covariates were controlled.*

Ecological, evolutionary & environmental sciences study design

All studies must disclose on these points even when the disclosure is negative.

Study description	*Briefly describe the study. For quantitative data include treatment factors and interactions, design structure (e.g. factorial, nested, hierarchical), nature and number of experimental units and replicates.*
Research sample	*Describe the research sample (e.g. a group of tagged Passer domesticus, all Stenocereus thurberi within Organ Pipe Cactus National Monument), and provide a rationale for the sample choice. When relevant, describe the organism taxa, source, sex, age range and any manipulations. State what population the sample is meant to represent when applicable. For studies involving existing datasets, describe the data and its source.*
Sampling strategy	*Note the sampling procedure. Describe the statistical methods that were used to predetermine sample size OR if no sample-size calculation was performed, describe how sample sizes were chosen and provide a rationale for why these sample sizes are sufficient.*
Data collection	*Describe the data collection procedure, including who recorded the data and how.*
Timing and spatial scale	*Indicate the start and stop dates of data collection, noting the frequency and periodicity of sampling and providing a rationale for these choices. If there is a gap between collection periods, state the dates for each sample cohort. Specify the spatial scale from which the data are taken*
Data exclusions	*If no data were excluded from the analyses, state so OR if data were excluded, describe the exclusions and the rationale behind them, indicating whether exclusion criteria were pre-established.*
Reproducibility	*Describe the measures taken to verify the reproducibility of experimental findings. For each experiment, note whether any attempts to repeat the experiment failed OR state that all attempts to repeat the experiment were successful.*
Randomization	*Describe how samples/organisms/participants were allocated into groups. If allocation was not random, describe how covariates were controlled. If this is not relevant to your study, explain why.*
Blinding	*Describe the extent of blinding used during data acquisition and analysis. If blinding was not possible, describe why OR explain why blinding was not relevant to your study.*

Did the study involve field work? ☐ Yes ☐ No

Field work, collection and transport

Field conditions	*Describe the study conditions for field work, providing relevant parameters (e.g. temperature, rainfall).*
Location	*State the location of the sampling or experiment, providing relevant parameters (e.g. latitude and longitude, elevation, water depth).*
Access & import/export	*Describe the efforts you have made to access habitats and to collect and import/export your samples in a responsible manner and in compliance with local, national and international laws, noting any permits that were obtained (give the name of the issuing authority, the date of issue, and any identifying information).*
Disturbance	*Describe any disturbance caused by the study and how it was minimized.*

Reporting for specific materials, systems and methods

We require information from authors about some types of materials, experimental systems and methods used in many studies. Here, indicate whether each material, system or method listed is relevant to your study. If you are not sure if a list item applies to your research, read the appropriate section before selecting a response.

Materials & experimental systems

n/a	Involved in the study	
☐	☒	Antibodies
☒	☐	Eukaryotic cell lines
☒	☐	Palaeontology and archaeology
☐	☒	Animals and other organisms
☐	☒	Human research participants
☒	☐	Clinical data
☒	☐	Dual use research of concern

Methods

n/a	Involved in the study	
☒	☐	ChIP-seq
☐	☒	Flow cytometry
☒	☐	MRI-based neuroimaging

Antibodies

Antibodies used

anti-FAPα (Invitrogen, BMS168, dilution 1:100),
anti-FAPα (Abcam, ab218164, dilution 1:100),
anti-Mfap4 (Thermo, PA5-24865, dilution 1:100),
anti-Sparcl1 (Santa Cruz, sc-514275,dilution 1:100),
anti-F4/80 (Cell Signaling Technology, #70076,dilution 1:500),
PerCP anti-CD3 (Biolegend, 317338, 5μl/test)
APC/Cyanine7 CD31 (Biolegend, 303120, 5μl/test),
APC anti-CD68 (Biolegend, 333810, 5μl/test),
PE/Cyanine7 anti-CD19 (Biolegend, 302216, 5μl/test),
PE Goat anti-mouse IgG (Biolegend, 405307, 5μl/test),
PE anti-human CD14 (Biolegend, 367104,5μl/test),
FITC anti-human CD90 (Thy1) (Biolegend, 328107, 5μl/test),
Dynabeads anti-CD19 (Invitrogen, 11143D, 50μl beads/2.5 x 10^7 cells/ml);
Dynabeads anti-CD2 (Invitrogen, 11159D, 50μl beads/2.5 x 10^7 cells/ml);
Dynabeads anti-CD14 (Invitrogen, 11149D, 50μl beads/2.5 x 10^7 cells/ml),
anti-SLC40A1 antibody (Abcam, ab78066,dilution 1:200),
anti-GCLM antibody (HuaBio, ET1705-87,dilution 1:500),
anti-GCLC antibody (HuaBio, ET1704-38,dilution 1:500),
anti-IκBα antibody (HuaBio, ET1603-6,dilution 1:500),
anti-phospho-IκBα antibody (HuaBio, ET1609-78,dilution 1:500),
anti-NF-kB p65 antibody (HuaBio, ET1603-12,dilution 1:500),
anti-SLC7A11 antibody (Abcam, ab175186,dilution 1:1000),
anti-c-JUN polyclonal antibody (Proteintech, 24909-1-AP,dilution 1:1000),
anti-ferritin rabbit monoclonal antibody (Abcam, ab75973,dilution 1:1000),
anti-GAPDH antibody (HuaBio, R1210-1,dilution 1:2000) ,
anti-β-actin antibody (HuaBio, 1210-2,dilution 1:2000).
4-hydroxynonenal (Abcam, ab48506,dilution 1:25),
anti-fibroblast activation protein (Abcam, ab53066,dilution 1:50),
anti-glutathione peroxidase 4 (Abcam, ab125066,dilution 1:50),
anti-GCLC (Abcam, ab53179,dilution 1:100),
anti-GCLM (Abcam, ab126704,dilution 1:50),
p-NF-κB p65 (Thermo Fisher, 44-711G,dilution 1:50).
8-OHdG (Abcam, ab48508,dilution 1:100)
VCAM-1 (Abcam, ab134047,dilution 1:500),
CD248 (Abcam, ab217535,dilution 1:200),
4-HNE (Abcam, ab46545,dilution 1:50),
TNFR1 neutralizing antibody (Sino Biological, 10872-R111,dilution 1:400),
TNFR2 neutralizing antibody (Sino Biological, 10417-R00N6,dilution 1:200).
rabbit IgG Isotype Control (Invitrogen, 31235,dilution 1:100),
mouse IgG Isotype Control (Invitrogen, 14-4714-82,dilution 1:1000)

Validation

All primary antibodies were confirmed or the species and application through the validation statement on the manufacturer's website and their use in the literature. We are providing here the list of antibodies used. anti-FAPα (Invitrogen, BMS168), validated by the company (https://www.thermofisher.com/cn/zh/antibody/product/FAP-Antibody-clone-F11-24-Monoclonal/BMS168) and by users (cited 4 times)
anti-FAPα (Abcam, ab218164), validated by the company (https://www.abcam.com/fibroblast-activation-protein-alpha-antibody-ab218164.html)
anti-Mfap4 (Thermo, PA5-24865), validated by the company (https://www.thermofisher.com/cn/zh/antibody/product/MFAP4-Antibody-Polyclonal/PA5-24865)

anti-Sparcl1 (Santa Cruz, sc-514275), validated by the company (https://www.scbt.com/p/sparcl1-antibody-g-5) and by users (cited 3 times)
anti-F4/80 (Cell Signaling Technology, #70076), validated by the company (https://www.cellsignal.com/products/primary-antibodies/f4-80-d2s9r-xp-rabbit-mab/70076) and by users (cited 63 times)

PerCP anti-CD3 (Biolegend, 317338), validated by the company (https://www.biolegend.com/en-us/products/percp-anti-human-cd3-antibody-9624?GroupID=GROUP28) and by users (cited 2 times)
APC/Cyanine7 CD31 (Biolegend, 303120), validated by the company (https://www.biolegend.com/en-us/products/apc-cyanine7-anti-human-cd31-antibody-6730) and by users (cited 11 times)
APC anti-CD68 (Biolegend, 333810), validated by the company (https://www.biolegend.com/en-us/products/apc-anti-human-cd68-antibody-6542) and by users (cited 14 times)
PE/Cyanine7 anti-CD19 (Biolegend, 302216), validated by the company (https://www.biolegend.com/en-us/products/pe-cyanine7-anti-human-cd19-antibody-1911) and by users (cited 31 times)
PE Goat anti-mouse IgG (Biolegend, 405307), validated by the company (https://www.biolegend.com/en-us/products/pe-goat-anti-mouse-igg-minimal-x-reactivity-1418) and by users (cited 26 times)
PE anti-human CD14 (Biolegend, 367104), validated by the company (https://www.biolegend.com/en-us/products/pe-anti-human-cd14-antibody-12011) and by users (cited 7 times)

FITC anti-human CD90 (Thy1) (Biolegend, 328107), validated by the company (https://www.biolegend.com/en-us/products/fitc-anti-human-cd90-thy1-antibody-4113?GroupID=BLG5826) and by users (cited 43 times)
anti-SLC40A1 antibody (Abcam, ab78066), validated by the company (https://www.abcam.com/slc40a1-antibody-ab78066.html) and by users (cited 15 times)

anti-GCLM antibody (HuaBio, ET1705-87), validated by the company (https://www.huabio.com/products/gclm-antibody-clone-jm93-61-recombinant-monoclonal-et1705-87)
anti-GCLC antibody (HuaBio, ET1704-38), validated by the company (https://www.huabio.com/products/gclc-antibody-clone-ja08-03-recombinant-monoclonal-et1704-38)

anti-IκBα antibody (HuaBio, ET1603-6), validated by the company (https://www.huabio.com/products/ikb-alpha-antibody-clone-sz00-07-recombinant-monoclonal-et1603-6) and by users (cited 4 times)
anti-phospho-IκBα antibody (HuaBio, ET1609-78), validated by the company (https://www.huabio.com/products/phospho-ikb-alpha-s32-antibody-clone-st53-05-recombinant-monoclonal-et1609-78)

anti-NF-kB p65 antibody (HuaBio, ET1603-12), validated by the company (https://www.huabio.com/products/nf-kb-p65-antibody-clone-sz10-04-recombinant-monoclonal-et1603-12) and by users (cited 5 times)
anti-SLC7A11 antibody (Abcam, ab175186), validated by the company (https://www.abcam.com/xct-antibody-epr82902-ab175186.html) and by users (cited 41 times)
anti-JUN polyclonal antibody (Proteintech, 24909-1-AP), validated by the company (https://www.ptglab.com/Products/JUN-Antibody-24909-1-AP.htm) and by users (cited 20 times)
anti-ferritin rabbit monoclonal antibody (Abcam, ab75973), validated by the company (https://www.abcam.com/ferritin-antibody-epr3004y-ab75973.html) and by users (cited 51 times)
anti-GAPDH antibody (HuaBio, R1210-1), validated by the company (https://www.huabio.com/products/gapdh-antibody-polyclonal-r1210-1) and by users (cited 8 times)
anti-β-actin antibody (HuaBio, 1210-2), validated by the company (https://www.huabio.com/products/beta-actin-antibody-clone-a2-f6-monoclonal-m1210-2) and by users (cited 51 times)
4-hydroxynonenal (Abcam, ab48506), validated by the company (https://www.abcam.com/4-hydroxynonenal-antibody-hnej-2-ab48506.html?productWallTab=Abreviews) and by users (cited 125 times)
anti-fibroblast activation protein (Abcam, ab53066), validated by the company (https://www.abcam.com/fibroblast-activation-protein-alpha-antibody-ab53066.html) and by users (cited 94 times)
anti-glutathione peroxidase 4 (Abcam, ab125066), validated by the company (https://www.abcam.com/glutathione-peroxidase-4-antibody-epncir144-ab125066.html) and by users (cited 165 times)
anti-GCLC (Abcam, ab53179), validated by the company (https://www.abcam.com/gclc-antibody-ab53179.html) and by users (cited 31 times)
anti-GCLM (Abcam, ab126704), validated by the company (https://www.abcam.com/GCLM-antibody-EPR6667-ab126704.html) and by users (cited 43 times)
p-NF-κB p65 (Thermo Fisher, 44-711G), validated by the company (https://www.thermofisher.com/cn/zh/antibody/product/Phospho-NFkB-p65-Ser529-Antibody-Polyclonal/44-711G) and by users (cited 19 times)
PE anti-CD45 (Biolegend, 304008), validated by the company (https://www.biolegend.com/en-us/products/pe-anti-human-cd45-antibody-708?GroupID=GROUP28) and by users (cited 20 times)
FITC anti-Collagen I (Sigma-Aldrich, FCMAB412F), validated by the company (https://www.sigmaaldrich.com/catalog/product/mm/fcmab412f?lang=en&region=US) and by users (cited 3 times)
8-OHdG (Abcam, ab48508), validated by the company (https://www.abcam.com/8-hydroxy-2-deoxyguanosine-antibody-n451-ab48508.html) and by users (cited 90 times)
VCAM-1 (Abcam, ab134047), validated by the company (https://www.abcam.com/VCAM1-antibody-EPR5047-ab134047.html) and by users (cited 186 times)

CD248 (Abcam, ab217535), validated by the company (https://www.abcam.com/tem1-antibody-ab217535.html)
4-HNE (Abcam, ab46545), validated by the company (https://www.abcam.com/4-Hydroxynonenal-antibody-ab46545.html) and by users (cited 387 times)
rabbit IgG Isotype Control (Invitrogen, 31235), validated by the company (https://www.thermofisher.cn/cn/zh/antibody/product/Rabbit-IgG-Isotype-Control/31235) and by users (cited 6 times)
mouse IgG Isotype Control (Invitrogen, 14-4714-82), validated by the company (https://www.thermofisher.cn/cn/zh/antibody/product/Mouse-IgG1-kappa-clone-P3-6-2-8-1-Isotype-Control/14-4714-82) and by users (cited 38 times)
TNFR1 / CD120a / TNFRSF1A Neutralizing Antibody, validated by the company (https://www.sinobiological.com/antibodies/human-tnfrsf1a-10872-r111) and by users (cited 4 times)
TNFR2 / CD120b / TNFRSF1B Neutralizing Antibody,validated by the company (https://www.sinobiological.com/antibodies/human-tnfr2-tnfrsf1b-10417-r00n6) and by users (cited 3 times)

Eukaryotic cell lines

Policy information about cell lines

Cell line source(s) | *State the source of each cell line used.*

Authentication | *Describe the authentication procedures for each cell line used OR declare that none of the cell lines used were authenticated.*

Mycoplasma contamination | *Confirm that all cell lines tested negative for mycoplasma contamination OR describe the results of the testing for mycoplasma contamination OR declare that the cell lines were not tested for mycoplasma contamination.*

Commonly misidentified lines (See ICLAC register)	*Name any commonly misidentified cell lines used in the study and provide a rationale for their use.*

Palaeontology and Archaeology

Specimen provenance	*Provide provenance information for specimens and describe permits that were obtained for the work (including the name of the issuing authority, the date of issue, and any identifying information).*
Specimen deposition	*Indicate where the specimens have been deposited to permit free access by other researchers.*
Dating methods	*If new dates are provided, describe how they were obtained (e.g. collection, storage, sample pretreatment and measurement), where they were obtained (i.e. lab name), the calibration program and the protocol for quality assurance OR state that no new dates are provided.*

☐ Tick this box to confirm that the raw and calibrated dates are available in the paper or in Supplementary Information.

Ethics oversight	*Identify the organization(s) that approved or provided guidance on the study protocol, OR state that no ethical approval or guidance was required and explain why not.*

Note that full information on the approval of the study protocol must also be provided in the manuscript.

Animals and other organisms

Policy information about studies involving animals; ARRIVE guidelines recommended for reporting animal research

Laboratory animals	DBA/1 male, 8-10 weeks.
Wild animals	The study did not involve wild animals.
Field-collected samples	The study did not involve samples collected from the field.
Ethics oversight	All animal procedures were performed in accordance with Laboratory Animal Ethics Committee of Air Force Medical University.

Note that full information on the approval of the study protocol must also be provided in the manuscript.

Human research participants

Policy information about studies involving human research participants

Population characteristics	No patients included in the study were being under corticosteroids treatment or second-line drug agents (methotrexate, sulfasalazine, or cyclosporin A) at the time of, and shortly before, the surgery (we set the standard as one month before the surgery). Clinicopathological measurements were recorded, including disease duration, composite 28-joint count Disease Activity Score (DAS28), erythrocyte sedimentation rate (ESR), C-reactive protein (CRP), and general health or the patient's global assessment of disease activity (using a 100-mm visual analog scale). All active RA and osteoarthritis (OA) patients met the clinical and radiographic criteria of the American College of Rheumatology. For immunohistochemistry and TSA-based immunofluorescent multiplex studies using human synovial biopsy tissue, the following patients were included: RA n = 26 (females, n = 14; range age 21-76, median age 48, IQR 39.5-54.3; high disease activity, n = 19, moderate disease activity, n = 7); OA n = 21 (females, n = 12;range age 45-72, median age 55, IQR 49.5-57). For the single-cell RNA sequencing, the following patients were included: RA n = 5 (females, n = 5; range age 38-62, mean age 49.8; high disease activity n = 5). For the measurement of MDA, 8-OH-dG and iron levels in joint fluid, the following RA patients were included: n = 20 (females, n = 16;range age 33-69,median age 53, IQR 50-61.3; high disease activity, n = 12; moderate disease activity, n = 8). For the isolation of synovial fibroblasts from joint fluid and circulating fibrocytes from peripheral blood mononuclear cells, the following active RA patients were included: n = 6 (females, n = 4; range age 31-68, mean age 48.3; high disease activity, n = 3; moderate disease activity, n = 3).
Recruitment	Patients with clinical synovitis met the clinical and radiographic criteria of the American College of Rheumatology and were not being treated with corticosteroids or second-line drug agents at the time of and shortly before the surgery were recruited to the study. No self-selection bias was present. All studies were approved by the Medical Ethics Committee and patients gave written, informed consent to participate.
Ethics oversight	Human specimen research was approved by the Medical Ethics Committee of the First Affiliated Hospital (Xijing Hospital) of the Fourth Military Medical University.

Note that full information on the approval of the study protocol must also be provided in the manuscript.

Clinical data

Policy information about clinical studies

All manuscripts should comply with the ICMJEguidelines for publication of clinical research and a completedCONSORT checklist must be included with all submissions.

Clinical trial registration	N/A

Study protocol	N/A
Data collection	N/A
Outcomes	N/A

Dual use research of concern

Policy information about dual use research of concern

Hazards

Could the accidental, deliberate or reckless misuse of agents or technologies generated in the work, or the application of information presented in the manuscript, pose a threat to:

No	Yes	
☐	☐	Public health
☐	☐	National security
☐	☐	Crops and/or livestock
☐	☐	Ecosystems
☐	☐	Any other significant area

Experiments of concern

Does the work involve any of these experiments of concern:

No	Yes	
☐	☐	Demonstrate how to render a vaccine ineffective
☐	☐	Confer resistance to therapeutically useful antibiotics or antiviral agents
☐	☐	Enhance the virulence of a pathogen or render a nonpathogen virulent
☐	☐	Increase transmissibility of a pathogen
☐	☐	Alter the host range of a pathogen
☐	☐	Enable evasion of diagnostic/detection modalities
☐	☐	Enable the weaponization of a biological agent or toxin
☐	☐	Any other potentially harmful combination of experiments and agents

ChIP-seq

Data deposition

☐ Confirm that both raw and final processed data have been deposited in a public database such as GEO.

☐ Confirm that you have deposited or provided access to graph files (e.g. BED files) for the called peaks.

Data access links<br>*May remain private before publication.*	*For "Initial submission" or "Revised version" documents, provide reviewer access links. For your "Final submission" document, provide a link to the deposited data.*
Files in database submission	*Provide a list of all files available in the database submission.*
Genome browser session<br>(e.g. UCSC)	*Provide a link to an anonymized genome browser session for "Initial submission" and "Revised version" documents only, to enable peer review. Write "no longer applicable" for "Final submission" documents.*

Methodology

Replicates	*Describe the experimental replicates, specifying number, type and replicate agreement.*
Sequencing depth	*Describe the sequencing depth for each experiment, providing the total number of reads, uniquely mapped reads, length of reads and whether they were paired- or single-end.*
Antibodies	*Describe the antibodies used for the ChIP-seq experiments; as applicable, provide supplier name, catalog number, clone name, and lot number.*
Peak calling parameters	*Specify the command line program and parameters used for read mapping and peak calling, including the ChIP, control and index files used.*

Data quality	*Describe the methods used to ensure data quality in full detail, including how many peaks are at FDR 5% and above 5-fold enrichment.*
Software	*Describe the software used to collect and analyze the ChIP-seq data. For custom code that has been deposited into a community repository, provide accession details.*

Flow Cytometry

Plots

Confirm that:

- [x] The axis labels state the marker and fluorochrome used (e.g. CD4-FITC).
- [x] The axis scales are clearly visible. Include numbers along axes only for bottom left plot of group (a 'group' is an analysis of identical markers).
- [x] All plots are contour plots with outliers or pseudocolor plots.
- [x] A numerical value for number of cells or percentage (with statistics) is provided.

Methodology

Sample preparation	Cells used in Flow Cytometry are cultured/uncultured human cells isolated from synovial biopsies, joint fluid or PBMC, described in Methods section. Cell death was analyzed by SYTOX Green staining followed flow cytometry. Samples were washed with PBS before flow cytometry. To analyze lipid peroxidation, cells were stained with BODIPY-C11 followed by flow cytometric analysis.
Instrument	Fortessa (BD Biosciences).
Software	BD FACSDiva Software was used to collect data, BD FACSDiva Software and Flowjo(v10.5.3) Software were used to analyze the data.
Cell population abundance	In flow cytometry analysis, up to 5,000 cells are examined.
Gating strategy	For cell death detection, unstained controls (PBS alone without staining) were used as negative control. The first plot (FSC-A vs. SSC-A) was drawn to exclude debris as they tend to have lower forward scatter levels. The second and third plots (SSC-W vs. SSC-H and FSC-W vs. FSC-H) are drawn to remove doublets from the analysis. Then a single parameter histogram was used to identify cells which would be positive for the dye. A gate boundary is made based on the unstained cells and the peaks of the histogram. For lipid ROS staining, samples without BODIPY-C11 was used as negative control. The figure exemplifying the gating strategy are provided in Supplementary information.

- [x] Tick this box to confirm that a figure exemplifying the gating strategy is provided in the Supplementary Information.

Magnetic resonance imaging

Experimental design

Design type	*Indicate task or resting state; event-related or block design.*
Design specifications	*Specify the number of blocks, trials or experimental units per session and/or subject, and specify the length of each trial or block (if trials are blocked) and interval between trials.*
Behavioral performance measures	*State number and/or type of variables recorded (e.g. correct button press, response time) and what statistics were used to establish that the subjects were performing the task as expected (e.g. mean, range, and/or standard deviation across subjects).*

Acquisition

Imaging type(s)	*Specify: functional, structural, diffusion, perfusion.*
Field strength	*Specify in Tesla*
Sequence & imaging parameters	*Specify the pulse sequence type (gradient echo, spin echo, etc.), imaging type (EPI, spiral, etc.), field of view, matrix size, slice thickness, orientation and TE/TR/flip angle.*
Area of acquisition	*State whether a whole brain scan was used OR define the area of acquisition, describing how the region was determined.*

Diffusion MRI ☐ Used ☐ Not used

Preprocessing

Preprocessing software	*Provide detail on software version and revision number and on specific parameters (model/functions, brain extraction, segmentation, smoothing kernel size, etc.).*
Normalization	*If data were normalized/standardized, describe the approach(es): specify linear or non-linear and define image types used for transformation OR indicate that data were not normalized and explain rationale for lack of normalization.*
Normalization template	*Describe the template used for normalization/transformation, specifying subject space or group standardized space (e.g. original Talairach, MNI305, ICBM152) OR indicate that the data were not normalized.*
Noise and artifact removal	*Describe your procedure(s) for artifact and structured noise removal, specifying motion parameters, tissue signals and physiological signals (heart rate, respiration).*
Volume censoring	*Define your software and/or method and criteria for volume censoring, and state the extent of such censoring.*

Statistical modeling & inference

Model type and settings	*Specify type (mass univariate, multivariate, RSA, predictive, etc.) and describe essential details of the model at the first and second levels (e.g. fixed, random or mixed effects; drift or auto-correlation).*
Effect(s) tested	*Define precise effect in terms of the task or stimulus conditions instead of psychological concepts and indicate whether ANOVA or factorial designs were used.*

Specify type of analysis: ☐ Whole brain ☐ ROI-based ☐ Both

Statistic type for inference (See Eklund et al. 2016)	*Specify voxel-wise or cluster-wise and report all relevant parameters for cluster-wise methods.*
Correction	*Describe the type of correction and how it is obtained for multiple comparisons (e.g. FWE, FDR, permutation or Monte Carlo).*

Models & analysis

n/a	Involved in the study
☐	☐ Functional and/or effective connectivity
☐	☐ Graph analysis
☐	☐ Multivariate modeling or predictive analysis

Functional and/or effective connectivity	*Report the measures of dependence used and the model details (e.g. Pearson correlation, partial correlation, mutual information).*
Graph analysis	*Report the dependent variable and connectivity measure, specifying weighted graph or binarized graph, subject- or group-level, and the global and/or node summaries used (e.g. clustering coefficient, efficiency, etc.).*
Multivariate modeling and predictive analysis	*Specify independent variables, features extraction and dimension reduction, model, training and evaluation metrics.*
